# Patient-reported outcome measurement in community-acquired pneumonia: feasibility of routine application in an elderly hospitalized population

**DOI:** 10.1186/s40814-019-0481-y

**Published:** 2019-07-27

**Authors:** Melanie A. Lloyd, Clarice Y. Tang, Emily J. Callander, Edward D. Janus, Amalia Karahalios, Elizabeth H. Skinner, Stephanie Lowe, Harin A. Karunajeewa

**Affiliations:** 1 Department of Physiotherapy, Western Health, St Albans, Victoria 3021 Australia; 2 Melbourne Medical School – Western Precinct, The University of Melbourne, St Albans, Victoria 3021 Australia; 3 Department of Physiotherapy, La Trobe University, Bundoora, Victoria 3000 Australia; 4 Department of Physiotherapy, Western Sydney University, Penrith, New South Wales 2751 Australia; 5 School of Medicine, Griffith University, Southport, Queensland 4215 Australia; 6 General Internal Medicine Unit, Western Health, Sunshine Hospital, St Albans, Victoria 3021 Australia; 7 Centre for Epidemiology and Biostatistics, Melbourne School of Population and Global Health, The University of Melbourne, Parkville, Victoria 3010 Australia; 8 Department of Physiotherapy, Melbourne School of Health Sciences, University of Melbourne, Parkville, Victoria 3010 Australia; 9 Department of Physiotherapy, School of Primary Care, Faculty of Medicine, Nursing and Health Sciences, Monash University, Frankston, Victoria 3199 Australia; 10 The Walter and Eliza Hall Institute of Medical Research, Parkville, 3052 Victoria Australia

**Keywords:** Outcome assessment, Pneumonia, Aged, Multimorbidity, Comorbidity, Inpatients

## Abstract

**Background:**

Community-acquired pneumonia (CAP) is a leading cause of morbidity and mortality worldwide, but few studies have evaluated the feasibility of routine patient-reported outcome measures (PROMs) in this illness. This study investigates the feasibility and limitations of three credible PROM instruments in a representative hospitalized cohort to identify potential barriers to routine application.

**Methods:**

A sample of multimorbid hospitalized subjects meeting a standardized CAP definition was recruited. Demographic and clinical data of those able and unable to participate in PROM assessment were compared. The EQ-5D-5L, CAP-Sym 18 Questionnaire, and Late-Life Function and Disability Instrument (LLFDI) were administered (via face-to-face interview) at admission and discharge and (via phone interview or mail) at 30 and 90 days post-discharge. Feasibility measures included the proportion of individuals able to participate in assessment, attrition rates, data completeness, and instrument completion times. Scores at admission and 30 days post-discharge were examined for association with age.

**Results:**

Of 82 subjects screened, 44 (54%) participated. Cognitive impairment (*n* = 12, 15%) commonly precluded participation. Seventeen (39%) participants were lost to follow-up by 90 days. Missing data at item level was negligible for all instruments, regardless of the mode of completion. Completion of the three instruments collectively in a face-to-face interview took a median of 17 min (IQ range 13–21) per participant. The burden of reported symptoms at admission was higher for younger participants aged 18–74 years (mean (standard deviation)) CAP-Sym 18 score at admission 34.2 (18.6) vs. 19.0 (11.3) for those aged ≥ 75 years.

**Conclusions:**

Routine application of PROMs can provide valuable information relating to multiple aspects of clinical recovery for individuals hospitalized with CAP. However, heterogeneous demographic characteristics and complex underlying health status introduce challenges to feasibility and interpretability of these instruments in this population.

**Trial registration:**

ClinicalTrials.gov, NCT02835040.

## Background

Community-acquired pneumonia (CAP) is not only a common cause of death in Australia [1], but also the third leading contributor to lost disability-adjusted life years worldwide, especially in the elderly population [2]. While it is often expected that patients with CAP will return to their pre-pneumonia baseline within a short period of time [3], many continue to suffer from significant deconditioning and loss of functional independence and wellbeing long after diagnosis [4, 5]. Misunderstanding of recovery may in part be due to the common outcome measures used to ascertain the effectiveness of treatment responses, such as time to clinical stability, adverse events, readmissions, and hospital length of stay [6–10], which largely support the interests of health providers rather than patients [11]. Consideration of patient-reported outcomes is vital to evaluate the impact of CAP on an individual’s perception of their wellbeing and function [12, 13]. This in return provides valuable information to health providers regarding how they can further improve treatment effectiveness and service delivery [11].

Developing a single PROM specific to CAP which encompasses all relevant patient-centered constructs is challenging due to the multisystem nature of the illness which, in addition to its impact on the respiratory organs, also frequently leads to the development of cardiac, gastrointestinal, and neurological complications [14, 15]. While popular generic PROMs, such as the EQ-5D-5L [16] can be used for people with CAP, these tools do not take into account disease-specific symptoms. Conversely, while existing disease-specific PROMs for CAP measure a broad range of symptoms relevant to the illness, they fail to consider the impact of existing co-morbidities in individuals with CAP [12, 17, 18], thereby perpetuating fragmented disease-centric, rather than person-centric, care [19]. Consideration of underlying chronic disease is particularly important due to the prevalence of CAP among older people, many of whom have pre-existing co-morbidities [20–22]. The use of a “modular” approach to PROMs [23], where an individualized combination of multiple short instruments is selected, may be useful to address the shortcomings of utilizing only a single disease-specific tool to capture all relevant aspects of patient recovery [12, 24]. This would allow consistent large-sample measurement of outcomes relevant to all patient groups, such as physical function and quality of life, using generic instruments, in addition to highly specific symptom scores tailored to one or multiple conditions as relevant.

While the use of PROMs provides valuable information about the impact of disease from the patient’s perspective, the limited uptake of PROMs in current CAP literature suggests potential challenges to their feasibility in this patient group. Resource limitations, including staff time required to perform assessments, and impediments to patient participation, such as sick, elderly patients unable or reluctant to complete survey instruments, may result in incomplete and biased data collection [25]. Advocates of the use of PROMs in CAP research have provided limited guidance regarding their practical application in representative patient cohorts [13, 24, 26], and feasibility of implementation needs further exploration. Additionally, all CAP-specific PROM instruments developed to date have been validated in population samples with a mean age under 70 years [17, 18, 27, 28], which raises questions regarding the generalizability of published statistical distributions of scores. This study therefore sought to examine the feasibility of routinely implementing three short PROM instruments (consistent with the “modular” approach presented above), each addressing a separate aspect of patient recovery: health-related quality of life (EQ-5D-5L English instrument [16]), physical function (Late-Life Function and Disability Instrument (LLFDI) [29]), and symptoms specifically related to CAP (CAP-Sym Questionnaire [17]). As none of these instruments have been explicitly validated in elderly hospitalized CAP populations, the data collected were examined to identify inconsistencies that may undermine interpretation of these instruments in the target patient group and prompt a need for further psychometric testing before integrating their use into routine practice.

## Methods

### Study design, setting, and participants

This prospective observational study was conducted at a tertiary metropolitan teaching hospital campus serving a population of approximately 700,000 in the western suburbs of Melbourne, Australia. This region has Australia’s highest proportion of non-English speaking migrants, with 58.4% of individuals speaking a language other than English at home [30]. Participants were prospectively recruited over 10 weeks between October and December 2016, as a nested “study within a trial” (SWAT) analysis for a larger study described elsewhere (trial registration: ClinicalTrials.gov, NCT02835040) [31].

All consecutive subjects hospitalized under general internal medicine units (managing the majority of adult CAP) who met a standardized definition of CAP [32], and were being actively treated, were screened for their ability to participate in PROM assessments. The following exclusion criteria were applied: (a) acute or chronic cognitive impairment impeding ability to provide informed consent, either documented in the subject’s medical record or based on clinical assessment by the treating medical team, (b) impaired conscious state, (c) receiving continuous ventilatory support, and (d) requiring language interpretation with medical assessments. Given the diversity of language backgrounds in the target population, and limitations on the availability of professional interpreting resources, cost constraints for this study meant that only English language assessments were conducted with data collected on language backgrounds to inform future work. Written informed consent was obtained from all individual participants.

### Instruments selected for pilot testing

The EQ-5D-5L is a simple questionnaire that allows an individual to rate their quality of life in the following five dimensions: mobility, self-care, usual activities, pain/discomfort, and anxiety/depression; with participants asked to rate each dimension on a five point scale [33]. The rating given to each dimension is later combined into a 5-digit number that describes the respondent’s health status. While yet to be validated specifically for individuals with CAP, the EQ-5D-5L has proven to be a valid and useful tool in appraising recovery, treatment response, and cost-effectiveness across multiple disease states [34, 35]. The UK utility index values and algorithm for health-related quality of life were used [36].

The LLFDI is an outcome measure that was specifically developed for community-dwelling older adults (age > 60 years) [29], has been validated in elderly inpatient populations [37], and the 32-item function component provides a self-report alternative to performance-based measures of function [38]. The “advanced lower limb” sub-score in particular has shown a high correlation with the performance of functional endurance tests [29]. Utilizing a 5-point scale, where “5 = no difficulty” while “1=cannot do the task at all,” participants were asked to rate their perceived level of difficulty in completing various tasks, with scores for all items aggregated and converted to a standardized scale [38]. Physical de-conditioning in elderly individuals (especially those hospitalized) is likely to be a major contributor to mortality and healthcare burden from CAP [39]. The LLFDI was chosen instead of other measures of dependence in activities of daily living (e.g., the Barthel Index [39] or Katz Index [5, 40], the function sub-score of the short form-36 (SF-36) [41], performance-based physical tests [42], or non-validated scores [15]) used in previous studies in CAP as it does not share the same shortcomings, such as ceiling effects, lack of specificity when function is the outcome of interest, impracticality in the target population, or a lack of credibility.

To augment the more generic EQ-5D-5L and LLFDI instruments, a CAP disease-specific tool was also sought. Although several symptom severity scores have been developed for and validated in CAP populations [17, 18, 27], the CAP-Sym Questionnaire was selected due to its brevity, clear and simple framework, and consistency with the 5-point rating scale design utilized in the EQ-5D-5L and LLFDI. The CAP-Sym has been validated in a large sample of individuals with CAP and is more responsive than the generic SF-36 [17].

All instruments, including scoring frameworks, were used in accordance with instructions from the instrument developers, and none were modified in any way. This meant that only the LLFDI could be used to quantify any aspects of pre-pneumonia health status, in this case, physical function, as both the EQ-5D-5L and CAP-Sym Questionnaire are designed to be rated only on the day of completion. Only English language versions of each instrument were used.

### Feasibility outcomes

Feasibility of the chosen instruments was assessed according to the following pre-specified measures.

1. Ability to participate in questionnaire-based assessment, as measured by the following:
  - Proportion willing and able to participate in the study in comparison to a recent large observational cohort benchmark (where recovery from CAP was assessed using PROMs as the primary outcome) of 32% [4]
  - Proportion meeting each individual exclusion criteria;
2. Missing data, measured by the following:
  - Participant attrition at each time-point during follow-up
  - Proportion of items marked incomplete in over 5% of questionnaire attempts [27]
3. Ease of use, measured by the following:
  - Time taken to complete each instrument face-to-face and via telephone
  - Reasons given by subjects or clinicians for difficulty or non-completion
4. Floor and ceiling effects calculated as percentage of participants recording the highest or lowest score on the scale—this value should not exceed 15% [43].

Measures of central tendency and variance for the obtained scores were compared between younger (18–74 years) and older (≥ 75 years) subjects to explore trends that may impact on the interpretation of scores from different age groups. The CAP-Sym and EQ-5D scores were compared between groups at admission to hospital and to measure recovery from admission to 90 days post-discharge. The LLFDI total and advanced lower limb scores were used to compare functional status pre-pneumonia to 30 days post-discharge.

### Data collection and management

Study data were collected and managed using REDCap electronic data capture tools [44] hosted at The University of Melbourne. Subjects were invited to participate in PROM assessments at four time points: admission to the ward, discharge from acute hospital stay, and 30 and 90 days post-discharge. Demographic and clinical data (age, sex, language status, residential status, comorbidities, and disease severity) were available for all CAP patients admitted during the SWAT analysis recruitment period via the main trial database. These data were used to identify differences in the characteristics of those individuals participating in the PROM feasibility study compared to those who did not. The EQ-5D-5L and CAP-Sym were completed at each time point. The LLFDI was completed at admission, where participants were asked to reflect on their functional performance on a typical day in the weeks prior to admission to hospital, and at 30 days post-discharge. Admission and discharge measures were completed in the hospital through face-to-face interview with a study investigator. Admission assessments were completed on the first day of hospital admission and discharge assessments on the last day of stay on the acute ward (i.e., immediately prior to discharge either home or to an interim sub-acute rehabilitation facility). Post-discharge PROMs were assessed verbally via phone. If participants were unable to be contacted by phone for five consecutive business days, or if they requested to complete questionnaires independently, copies of instruments were provided via post. If forms were not returned to the study coordinator within 30 days of posting, the participant was considered lost to follow-up for post-discharge outcomes.

### Statistical analysis

Descriptive statistics are reported as mean (standard deviation (SD)) or median (inter-quartile range (IQR)) for normally and non-normally distributed continuous variables, respectively, and counts (percentages) for categorical variables. Statistical analyses were completed using Stata version 14.2 (StataCorp, College Station, Texas, USA).

## Results

**Fig. 1:**
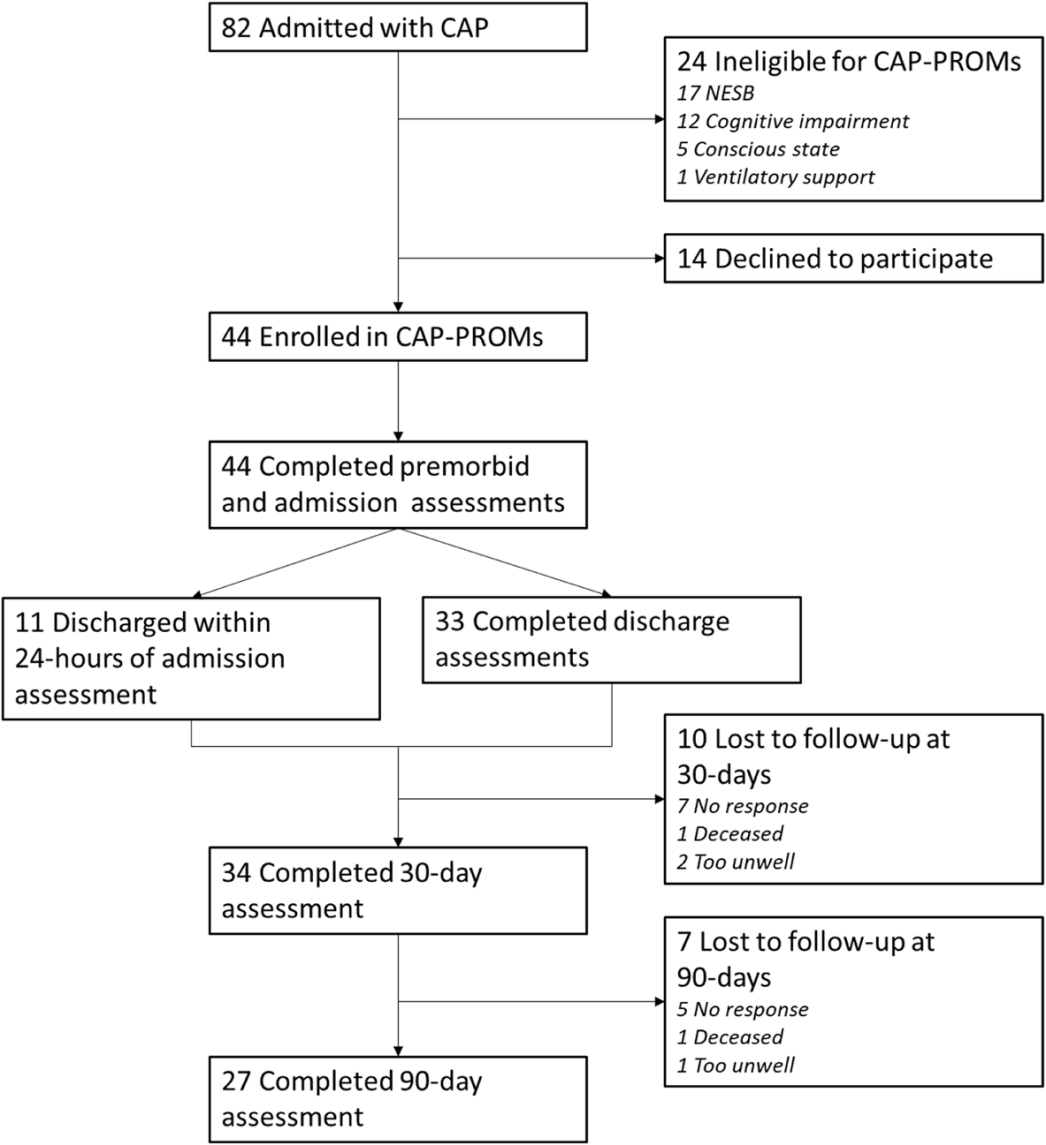
Subject flow through the study. More than one reason for ineligibility may apply to a single participant; hence, the tally does not total 24. Abbreviations: CAP community-acquired pneumonia, NESB non-English speaking background, PROMs patient-reported outcome measures

Of the 82 CAP patients admitted during the recruitment period, 24 (29%) were unable to participate most commonly due to either limited English (*n* = 17, 21%) and/or cognitive impairment (*n* = 12, 15%), and a further 14 (17%) declined to participate (Fig. 1). All 44 participants successfully completed required inpatient assessments, with 10 (23%) and 17 (39%) lost to follow-up at 30 and 90 days, respectively.

Subjects who participated in PROM assessments were more likely to be younger (median age 73.5 [IQR 63.0–80.0] vs. 77.0 [IQR 72.0–84.0] years for non-participants), living independently in the community (91% vs. 82%), and able to walk independently (98% vs. 79%) (Table 1). As expected given study exclusion criteria, non-participants had a higher proportion of diagnosed dementia (18% vs. 2%) and acute confusion (26% vs. 2%), admission to intensive care (11% vs. 0%), and a non-English primary language (47% vs. 11%) compared to those that participated.

**Table 1:** Baseline characteristics, disease severity, and recovery of patients presenting with CAP: October–December 2016

		Consenting participants*	Declined participation or excluded*
		(*n* = 44)	(*n* = 38)
Demographics	Age (years)	73.5 [63.0–80.0]	77 [72.0–84.0]
	Males	23 (52.3%)	23 (60.1%)
	NESB	5 (11.4%)	18 (47.4%)
	Aged care resident	4 (9.1%)	7 (18.4%)
	Lives alone	3 (6.8%)	10 (26.3%)
Premorbid health status	CCMI ≥ 7	14 (31.8%)	15 (39.5%)
	Anxiety/depression	7 (15.9%)	7 (18.4%)
	Chronic pulmonary disease	26 (59.1%)	18 (47.4%)
	CCF	9 (20.5%)	9 (23.7%)
	Diabetes	16 (36.4%)	16 (42.1%)
	Dementia	1 (2.2%)	7 (18.4%)
	Malnutrition (MST score ≥ 2)	11 (25.0%)	15 (39.4%)
	Walks without assistance†	43 (97.7%)	30 (78.9%)
Disease severity and complications	CURB-65‡ ≥ 3	19 (43.2%)	19 (50.0%)
	ICU admission	0 (0%)	4 (10.5%)
	Acute cardiac event	5 (11.4%)	8 (21.1%)
	Exacerbation CCF	7 (15.9%)	5 (13.2%)
	Acute confusion	1 (2.2%)	10 (26.3%)
Recovery	LOS (days)	4 [3–5]	4 [3–6]
	30-day readmission	3 (6.8%)	6 (15.8%)
	In hospital mortality	0 (0%)	1 (2.6%)
	Death within 30 days	1 (2.3%)	3 (7.9%)

*Abbreviations*: *CAP* community-acquired pneumonia, *CCF* congestive cardiac failure, *CCMI* Charlson Comorbidity Index, *LOS* length of hospital stay, *MST score* Malnutrition Screening Tool, *NESB* non-English speaking background (patient may or may not also be proficient in English)

^*^All data presented are median (interquartile range) or count (percentage)

†Walks without assistance: may use a gait aid but does not require the assistance of another person

‡CURB-65 score comprised of confusion, urea > 7 mmol/L, respiratory rate ≥ 30 per minute, blood pressure < 90 mmHg systolic, ≤ 60 mmHg diastolic, and age ≥ 65 years

The CAP-Sym and EQ-5D-5L Questionnaires had the shortest completion times (face-to-face median [IQR] 4 [2.5–5] and 3 [2–5] min, respectively) while the LLFDI contains a larger number of items and therefore took longer to complete (10 [6–11] min) (Table 2). A face-to-face assessment requiring completion of all three measures took an aggregate median of 17 min [IQR 13–21], and there was very little difference compared to completion via telephone (16 min [IQR 12–20]). Missing data at instrument level only influenced assessments completed via phone or mail (Table 2), primarily due to an inability to contact, or non-response of participants. At item level, missing data were negligible for all instruments. Hearing impairment, subject unavailability due to ongoing ill health, and poor recall were common difficulties reported by research assistants during the PROM assessments. Only the EQ-5D-5L utility index displayed ceiling effects outside the acceptable 15% range.

**Table 2:** Patient-reported outcome measure completion time and missing data by instrument and mode of collection

			EQ-5D	LLFDI	CAP-Sym 18
Completion time (minutes) (median, [IQ range])	Admission face-to-face (*n = 44*)		3 [2–5]	10 [6–11]	4 [2.5–5]
	30-day phone (*n = 34*)		3 [2–5]	10 [7–13]	3 [2–5]
Missing data (*n/N* * (%))	Instrument level	Face-to-face	0/87 (0%)	0/44 (0%)	0/87 (0%)
		Phone	20/78 (25.6%)	10/44 (22.7%)	20/78 (25.6%)
		Mail	12/20 (60.0%)	7/10 (70.0%)	14/20 (70.0%)
	Item level†: No. of items with > 5% missing values	Face-to-face	0/6 (0%)	0/32 (0%)	0/18 (0%)
		Phone	0/6 (0%)	2/32 (6.25%)	0/18 (0%)
		Mail	0/6 (0%)	0/32 (0%)	0/18 (0%)

*Abbreviations*: *CAP-Sym 18* CAP-Symptom Questionnaire (18-item version), *EQ-5D* EuroQol Questionnaire, *IQ* interquartile, *LLFDI* Late Life Function and Disability Instrument

^*^For instrument level data, the denominator represents the total number of occasions the instrument was attempted via that mode. Numbers differ between instruments because the LLFDI was only completed on admission and at 30 days

†For item level data, the denominator represents the number of items in that instrument

**Fig. 2:**
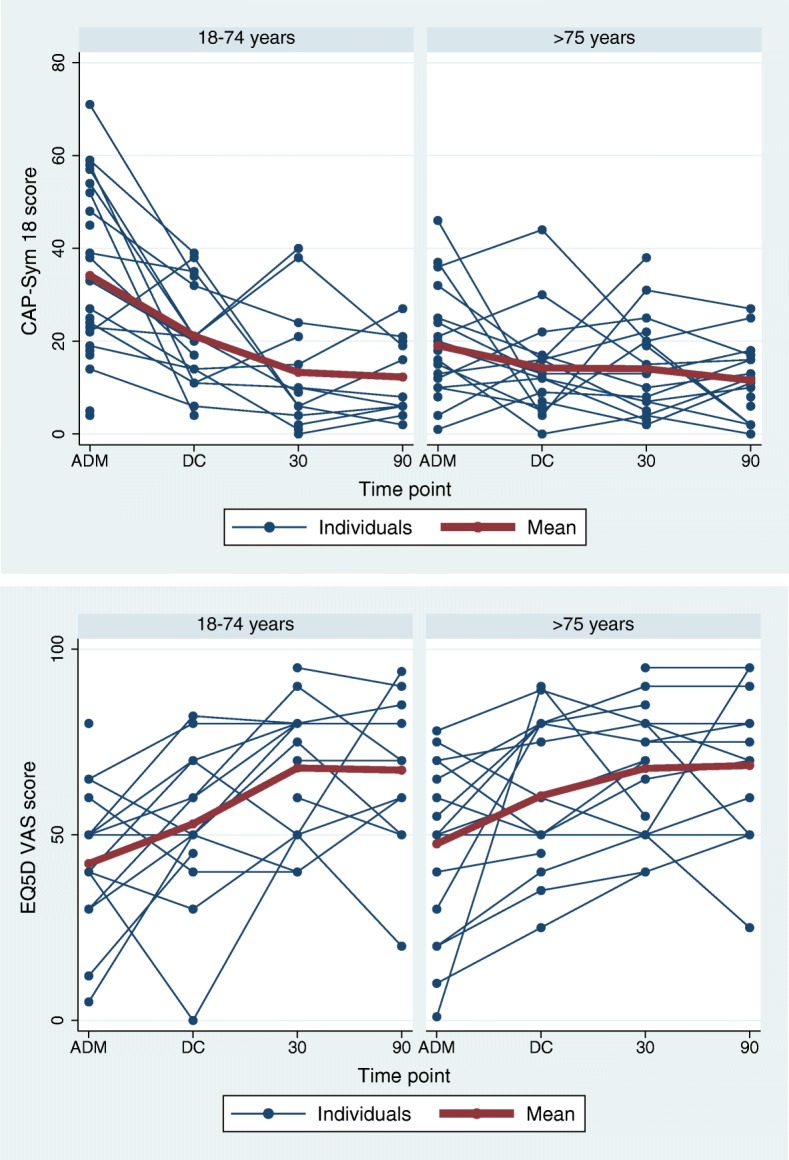
Change in CAP-Sym 18 and EQ-5D visual analog scores over time according to age group. *Instrument scoring (y-axis):* CAP-Sym18 score: CAP-Symptom Questionnaire score, consists of 18-items rated 0–5 where 0 has not experienced symptom and 5 extremely bothered by that symptom, total possible score between 0 and 90; EQ-5D VAS score: EQ-5D-5L visual analog scale score, rated as 0–100 where 0 is the worst health imaginable and 100 the best health imaginable. *Time points (x-axis):* ADM admission assessment conducted on first day of hospitalization, DC discharge assessment conducted on day of discharge, 30: 30-day assessment conducted at 30-days post discharge, 90: 90-day assessment conducted at 90 days post discharge

There was a trend for younger participants (aged 18–74 years) to report a higher burden of symptoms at admission when compared to those aged ≥ 75 (mean (SD) CAP-Sym 18 admission score 34.2 (18.6) vs. 19.0 (11.3)). (Table 3 and Fig. 2). At discharge, the burden of symptoms had reduced for both age groups (CAP-Sym 18 discharge score for those aged 18–74 years 21.1 (11.3) and ≥ 75 years 14.3 (10.2)). There was a small decrease in the ability to perform functional endurance tasks at 30 days for participants aged ≥ 60 years (LLFDI advanced lower limb score median [IQR] 33.1 [27.7–47.8] pre-morbid vs. 33.1 [11.35–47.8] 30 days).

**Table 3:** Difference in patient-reported outcome measure scores at admission between older and younger participants

Instrument	Age groups	
	18–74 years (*n* = 23) (mean (SD) or median (IQR))	≥ 75 years (*n* = 21) (mean (SD) or median (IQR))
CAP-Sym 18 score	34.2 (18.6)	19.0 (11.3)
EQ-5D VAS score	42.3 (18.8)	47.6 (21.3)
EQ-5D-5L index	0.54 [0.28–0.84]	0.68 [0.35–0.88]

*Abbreviations*: *CAP-Sym 18* CAP-Sym Questionnaire 18-item score, *CI* confidence interval, *EQ-5D VAS* EuroQol Quality of Life Questionnaire visual analog score, *EQ-5D-5L index score* EuroQol 5-dimension 5-level Questionnaire, *IQR* interquartile range, *PROMs* patient-reported outcome measure

^*^Age 18–74 years is the reference group

## Discussion

The results of this study highlight some of the challenges of administering PROMs to patients hospitalized for CAP and may explain why the utilization of PROMs, particularly as primary end-points, has remained limited in CAP research. Language barriers, impaired cognition, and severity of illness resulted in the exclusion of 29% of the total subjects admitted with CAP during the study period from PROM assessment. Additionally, hearing and visual impairment complicated the participant consent and PROM assessment process in a number of cases. While the CAP-Sym, LLFDI, and EQ-5D-5L are currently available in 13, 3, and 150 language versions, respectively, the feasibility of these PROMs has yet to be tested among populations from diverse cultural backgrounds. Significant interpreting resources will be required to facilitate the utilization of PROMs in health settings similar to ours.

Other than language difficulties, acute and chronic cognitive impairment also posed another challenge in administering PROMs in the hospital setting. While the self-reported quality of life instruments developed specifically for dementia have shown promise [45], self-report is not always viable for those with cognitive impairment, and other available options for outcome measurement must be considered for this group. This also applies to the use of PROMs in individuals with acute illness leading to impaired conscious state or other severe symptoms that impede the ability to participate in the questionnaire-based assessment. Time constraints and concentration span hinder the completion of long or multiple questionnaires, forcing researchers and clinicians to prioritize the types of information that is sought. In our study, while the individual PROM questionnaires were completed quickly, in aggregate, the three instruments took over 15 min in the majority of subjects, emphasizing the resource-intensive nature of this type of outcome measure.

Challenges in conducting a longitudinal cohort study to monitor changes in PROMs have also been identified in this study. Despite concerted efforts to follow-up participants via both phone and mail, subject attrition was around 40% at 90 days. This attrition rate, though not unprecedented [46], is higher than other large observational studies in CAP [4] and may be a direct result of the study’s inclusive recruitment strategy. Routine follow-up post-discharge is difficult in this population due to inherent challenges associated with ongoing illness in the frail elderly and the increased dependence on others for care. While representative recruitment enables the generalization of results to a broader population, a risk of higher participant attrition during longitudinal follow-up is a trade-off that must be considered. Providing flexible options for mode of completion to maximize longitudinal participation must be balanced against the risk of bias introduced by different modes [47]. Future research could consider alternative methods to reduce attrition, such as facilitation via a family member, carer or primary care clinician, or the use of technology to provide reminders and internet-based options for instrument completion.

Other than identifying the challenges faced when administering PROMs, this study also highlighted important considerations to be taken when interpreting PROM data in CAP populations. Consistent with previous studies [18, 48], older individuals tended to report less bothersome symptoms at admission and reported less improvement 30 days after discharge. Possible reasons for this trend include (i) younger individuals generally need to be more symptomatic relative to elderly to be hospitalized, whereas the threshold for admission in the elderly may be much lower; (ii) age-related immunologic changes may dampen systemic reactions to infection such as fever and sweating [18], and (iii) older individuals with chronic illness may be accustomed to poor health meaning the marginal impact of an acute episode is reduced. It is therefore possible that outcome scores may be less responsive to change in elderly, multimorbid cohorts due to the high burden of chronic illness. This may also explain the lower CAP-Sym scores at admission reported in this study when compared to others conducted in younger cohorts with less burden of chronic disease [49, 50].

The results of this study also demonstrated that, for a number of participants, endurance during physical tasks was reduced even a month after discharge from hospital. It may be hypothesized, based on results of this and other studies [5, 51], that long-term ongoing decrease in overall health status reported by CAP patients is linked to fatigue and reduced endurance for activities of daily living, rather than overall burden of symptoms which appear to return quickly to baseline [3]. Further research is required to fully investigate the role of impaired endurance in poor long-term outcomes from CAP and identify effective interventions to address poor exercise tolerance.

This study is the first study to the author’s knowledge to investigate the feasibility of implementing PROMs for patients hospitalized with CAP, though it has several important limitations. Due to the convenience sampling approach employed within a larger trial, the overall sample size was small. Results of the analysis should therefore be considered exploratory, though they provide a useful starting point for researchers considering applying PROMs in hospitalized elderly populations. The limited sample size was compounded by a lack of access to interpreting services which precluded measurement of PROM feasibility in patients from non-English speaking backgrounds. Results of the study can therefore only be generalized to English-speaking patient groups. Finally, while levels of attrition were in themselves an interesting outcome of this study, high numbers lost to follow-up precluded statistical analysis of the 90-day outcomes due to the risk of bias introduced by missing data in an already small sample.

## Conclusions

A modular approach to PROMs, comprising routine application of three short instruments (EQ5D, LLFDI, and CAP-Sym), can provide valuable information relating to multiple aspects of clinical recovery for individuals hospitalized with CAP. However, the heterogeneous characteristics, acuity of illness, and complex underlying health status of this population preclude the participation of a significant proportion of individuals in PROM assessment, introducing challenges to feasibility and interpretability of these instruments. The exclusion of individuals from diverse language backgrounds and with cognitive impairment from PROM assessment is a particular concern, and high rates of attrition also affected the feasibility of longitudinal assessment with these instruments.

## Data Availability

The datasets generated and analyzed during the current study are available from the corresponding author on reasonable request.

## Abbreviations

CAP: Community-acquired pneumonia
CAP-Sym: CAP-symptom questionnaire
CURB-65: Comprised of confusion (acute), urea > 19 mg/dL or > 7 mmol/L, respiratory rate ≥ 30 per minute, Systolic BP < 90 mmHg or diastolic BP ≤ 60 mmHg, age ≥ 65 years
EQ-5D-5L: EuroQol 5-dimension 5-level health-related Quality of Life Questionnaire
HREC: Human Research Ethics Committee
IQR: Interquartile range
LLFDI: Late-Life Function and Disability Instrument
LOS: Length of stay
MST: Malnutrition Screening Tool
NESB: Non-English speaking background
PROM: Patient-reported outcome measure
RCT: Randomized controlled trial
REDCap: Research Electronic Data Capture
SF-36: Rand Short Form 36 Questionnaire
UK: United Kingdom
US: United States of America
VAS: Visual analog scale
